# Tailoring the Preformed Solid Electrolyte Interphase in Lithium Metal Batteries: Impact of Fluoroethylene Carbonate

**DOI:** 10.1021/acsami.3c12797

**Published:** 2023-11-08

**Authors:** Dominik Weintz, Sebastian P. Kühn, Martin Winter, Isidora Cekic-Laskovic

**Affiliations:** † Helmholtz-Institute Münster (IEK-12), Forschungszentrum Jülich GmbH, Corrensstrasse 48, 48149 Münster, Germany; ‡ MEET Battery Research Center, University of Muenster, Corrensstraße 46, 48149 Muenster, Germany

**Keywords:** lithium metal, lithium metal pretreatment, solid electrolyte interphase, film-forming additive, fluoroethylene carbonate

## Abstract

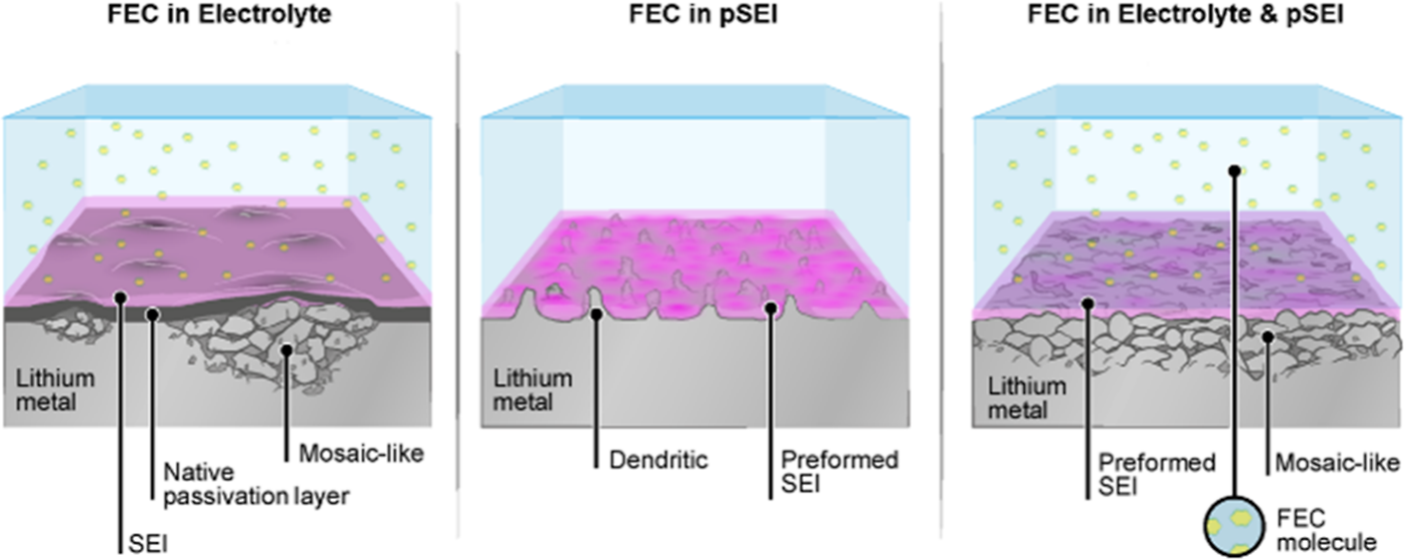

The film-forming electrolyte additive/co-solvent fluoroethylene carbonate (FEC) can play a crucial role in enabling high-energy-density lithium metal batteries (LMBs). Its beneficial impact on homogeneous and compact lithium (Li) deposition morphology leads to improved Coulombic efficiency (CE) of the resulting cell chemistry during galvanostatic cycling and consequently an extended cell lifetime. Herein, the impact of this promising additive/co-solvent on selected properties of LMBs is systematically investigated by utilizing an in-house developed lithium pretreatment method. The results reveal that as long as FEC is present in the organic carbonate-based electrolyte, a dense mosaic-like lithium morphology of Li deposits with a reduced polarization of only 20 mV combined with a prolonged cycle life is achieved. When the pretreated Li electrodes with an FEC-derived preformed SEI (pSEI) are galvanostatically cycled with the FEC-free electrolyte, the described benefits induced by the additive are not observable. These results underline that the favorable properties of the FEC-derived SEI are beneficial only if there is unreacted FEC in the electrolyte formulation left to constantly reform the interphase layer, which is especially important for anodes with high-volume changes and dynamic surfaces like lithium metal and lithiated silicon.

## 1 Introduction

The increasing demand for energy-dense storage systems mainly caused by the electrification of the transportation sector directed intensified research toward the further development of lithium-based batteries.^1^ Replacing the graphite-based anode with metallic lithium is one of the most promising approaches to considerably increase the energy density of the resulting battery chemistry.^2−5^ However, the employment of a “host-less” metallic electrode is accompanied by many challenges, such as infinite volume changes and resulting inhomogeneous lithium deposition, which to this point denied rechargeable lithium metal batteries in liquid electrolyte a broad industrial commercialization.^3,6−8^ Furthermore, the high reactivity of lithium metal associated with the low reduction potential of just −3.04 V (vs standard hydrogen electrode) leads to decomposition reactions as soon as it comes in contact with the electrolyte formulation, as all common liquid electrolyte components are unstable at such potentials.^9−11^ The resulting decomposition products form a solid electrolyte interphase (SEI) that ideally protects the corresponding electrode from further unwanted side reactions while still being able to conduct Li^+^ ions sufficiently.^11−16^ However, the large volume changes of lithium result in substantial internal stress accumulation, eventually causing cracking and reformation of the SEI during galvanostatic cycling.^7,15,17^ This continuous consumption of the electrolyte and active lithium naturally decreases the Coulombic efficiency (CE), which leads to a rapidly declining capacity retention and thus an ultimately early cell death.^15,18^

The use of functional electrolyte additives is regarded as one of the most favorable and cost-effective approaches to enhance the properties of the SEI and thereby improve the electrochemical performance of related lithium metal batteries (LMBs).^19−21^ The already commercialized film-forming additive fluoroethylene carbonate (FEC) has been shown to not only improve the galvanostatic cycling performance in graphite-based batteries^22^ but also enhance the cell lifetime with electrodes suffering from high changes in volume such as silicon^23−26^ and lithium metal.^27−31^ Zhang et al. revealed a more uniform lithium deposition accompanied by a significantly improved CE with the addition of FEC in an organic carbonate-based electrolyte.^27^ Promoting a thin and robust LiF-rich SEI combined with faster reaction kinetics facilitates a homogeneous current distribution during galvanostatic cycling, thus reducing stress-build up in the SEI.^32,33^ However, the effects of FEC on the interfacial and interphasial properties in LMBs have so far been investigated with pristine lithium (pLi) that is naturally covered by a native passivation layer (NPL), mainly composed of lithium carbonate, hydroxide, and oxide.^34−38^ In our previous publication, we reported a novel lithium pretreatment method to obtain an as-defined preformed SEI (pSEI) that solely consists of electrolyte decomposition products and enables the investigation of interfacial/interphasial properties on lithium in the absence of a NPL.^39^

In this study, the pretreatment method was utilized to investigate the impact of various FEC concentrations in the resulting electrolyte formulation and during the pretreatment of the lithium electrodes on the properties of the as-defined pSEI and its implications on the galvanostatic cycling performance in symmetric Li||Li as well as in a full-cell setup with a state-of-the-art LiNi_0.8_Mn_0.1_Co_0.1_O_2_ (NMC811) electrode. Analogous to the electrochemically derived SEI on lithium, the presence of FEC during the pretreatment also induces a LiF-rich passivation layer. The positive effects associated with this additive/co-solvent, including a dense mosaic-like Li deposition morphology, less resistive interphase, and prolonged lifetime of lithium metal-based cells, were further enhanced by employing a pretreated lithium electrode. However, the absence of FEC in the electrolyte diminishes the positive effects of the LiF-rich pSEI and resulting cells have shown similar characteristics as cells without the FEC-derived pSEI. Especially during galvanostatic cycling of symmetric Li||Li and NMC811||Li full-cells, the importance of FEC present during cycling became evident as the cycle life of the cell correlated with the amount of FEC in the considered electrolyte formulation.

## 2 Experimental Section

### 2.1 Materials

The lithium metal rods (Ø13 mm) used to prepare the Li metal electrodes were purchased from Sigma-Aldrich and used without purification. The NMC811 electrodes (CUSTOMCELLS) used for the full-cell setup with an aerial capacity of 1.0 mA h cm^–2^ were dried under vacuum at 120 °C for 24 h before use. The considered electrolyte formulations (E-Lyte Innovation) are listed in Table 1.

**Table 1:** Electrolyte Formulations Used in This Study

name	electrolyte formulation
baseline electrolyte (BE)	1.2 M LiPF_6_ in EC/EMC (3:7)
3FEC	1.2 M LiPF_6_ in EC/EMC (3:7) + 3.14 wt % FEC
6FEC	1.2 M LiPF_6_ in EC/EMC (3:7) + 6.09 wt % FEC
9FEC	1.2 M LiPF_6_ in EC/EMC (3:7) + 8.87 wt % FEC
12FEC	1.2 M LiPF_6_ in EC/EMC (3:7) + 11.48 wt % FEC

All considered materials were stored in an argon-filled glovebox (MBraun Labmaster, CO_2_ < 0.1 ppm, H_2_O < 0.1 ppm, O_2_ < 0.1 ppm, and N_2_ < 5 ppm) with a built-in nitrogen removal system.

### 2.2 Preparation of Lithium Metal Electrodes

The Li electrodes (thickness: 200 μm) were prepared with in-house built cutting and pressing devices according to our previous publication.^39^ For this purpose, a lithium rod was placed in the cutting device, and the surrounding bowl was filled with the respective electrolyte solution. After cutting the lithium rod utilizing two tungsten wires, the lithium discs and electrolyte solution were transferred into the pressing device, placed between two copper sheets, and pressed down until the targeted thickness of 200 μm was reached. The pretreated electrodes are noted as electrolyte@Li and the pristine lithium electrodes (pLi) were generated using the same lithium rod without an electrolyte present during the preparation of the electrodes. Furthermore, Mylar foil was used for the pressing process instead of copper sheets.

### 2.3 Surface Characterization

SEM measurements were performed in a Carl Zeiss Auriga Modular Crossbeam workstation with a Schottky field emission gun and a Gemini column as the electron source. The images were taken at an accelerating voltage of 3 kV and a working distance of 3.0 mm.

For the *post mortem* surface morphology analysis, the electrodes were disassembled from the coin cell setup, gently washed with 1 mL of EMC, and dried in a vacuum for 30 min.

For the XPS measurements, a photoelectron spectrometer of the type “K-Alpha” by Thermo VG Scientific was used with monochromatic Al Kα X-rays (*h*ν = 1486.6 eV) and a chamber pressure of 2 × 10^–9^ mbar.

### 2.4 Electrochemical Measurements

All electrochemical measurements were performed in a two-electrode configuration. Lithium stripping/plating was studied in a symmetric coin cell setup (type CR2032). For this purpose, two freshly prepared Li metal electrodes were combined with one Celgard 2500 separator soaked with 30 μL electrolyte. Galvanostatic cycling was conducted with a MACCOR battery cycler (MACCOR Series 4000) at 20 °C with a constant current density of 0.5 mA cm^–2^ and 1 h stripping/plating steps (0.5 mA h cm^–2^). The impedance measurements were carried out using a Bio-Logic VMP3 workstation with the same cycling parameters. After every 5 charge/discharge cycles, the impedance was recorded over a frequency range of 1 mHz to 100 MHz and fitted using an equivalent circuit selection of *R*_El_ + *R*_SEI_/*C*_SEI_ + *R*_CT_/*C*_CT_. For the galvanostatic cycling experiments in the full-cell setup, NMC811 electrodes (1.0 mA h cm^–2^) were used and the corresponding cells galvanostatically cycled at the constant current of 0.5 mA cm^–2^ (equivalent to ∼0.5C) in the voltage range from 4.2 to 3.0 V.

## 3 Results and Discussion

### 3.1 Characterization of the Solid Electrolyte Interphase Formed on the Lithium Metal Anode

**Figure 1:**
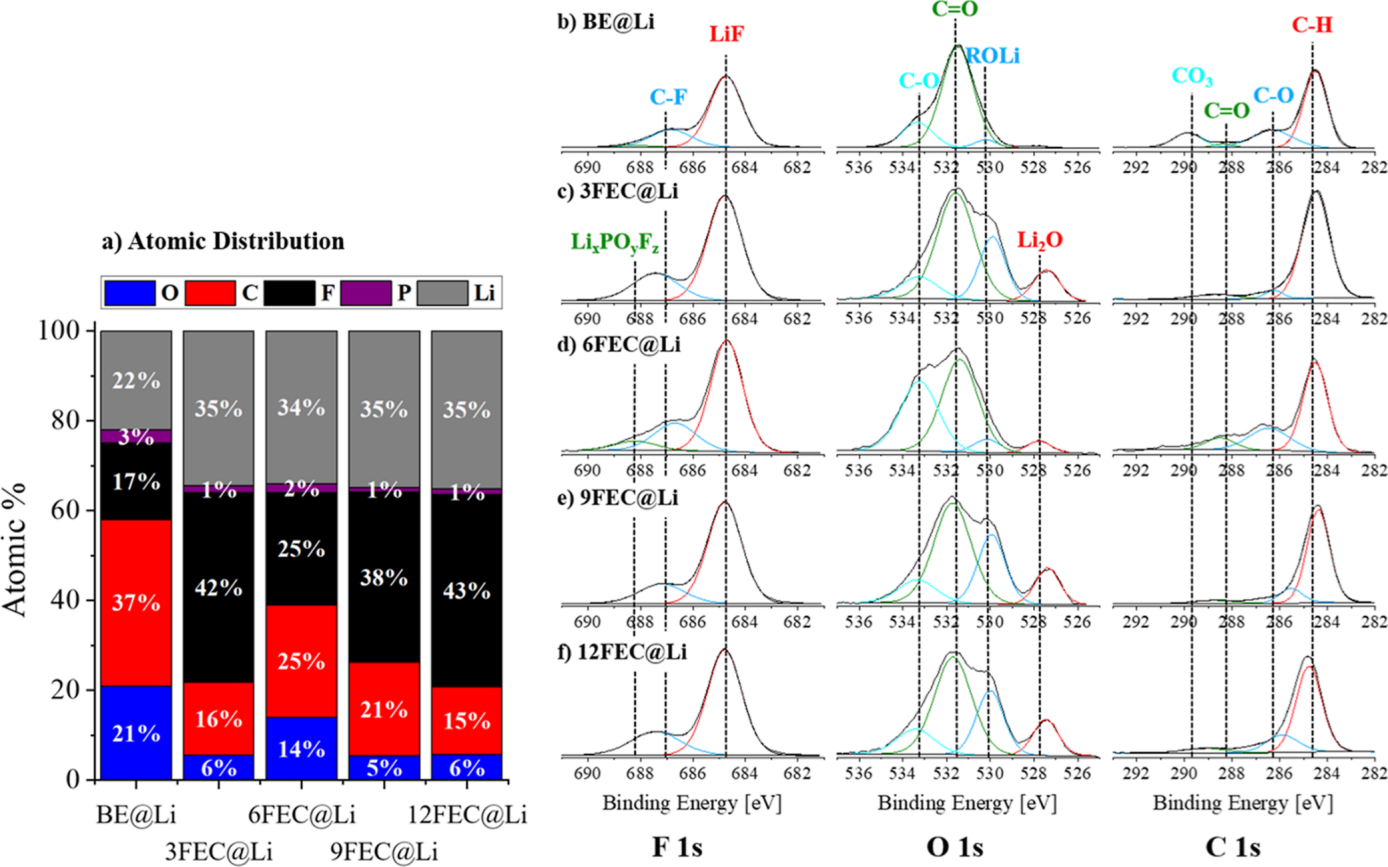
XPS analysis of the pSEI on pretreated electrodes. (a) Atomic distribution of O, C, F, P, and Li; (b–f) chemical resonance with the focus on F 1s, O 1s, and C 1s of (b) BE@Li, (c) 3FEC@Li, (d) 6FEC@Li, (e) 9FEC@Li, and (f) 12FEC@Li.

The chemical composition of the lithium anode surfaces was characterized and analyzed via X-ray photoelectron spectroscopy (XPS) to gain insights into the pSEI obtained from considered electrolyte formulations (Figure 1). The presence of FEC in the electrolyte solution during the electrode pretreatment results in a considerable increase of the relative fluorine content in the pSEI from 17% for the FEC-free BE@Li up to 43% for 12FEC@Li which can be mainly attributed to pronounced LiF formation (684.8 eV) in the F 1s spectra.^9,40^ In contrast to the assumption, the fluorine content does not increase with the FEC concentration during pretreatment as 3FEC induced a higher fluorine content in the pSEI with 42% compared to 6FEC (25%) and 9FEC (38%). The higher fluorine concentrations of the FEC-induced pSEIs are accompanied by a substantial decrease in both the oxygen and carbon content and according to the decreased CO_3_^–^ (290 eV) and C=O– resonance (523 eV), a formation of Li_2_CO_3_ is therefore less pronounced in the presence of FEC.^13,40^ Additionally, an abundance of alcoholates (ROLi, 530 eV) and low amounts of Li_2_O (528 eV) are present in the FEC-derived pSEIs, which were also suggested as FEC decomposition intermediates by complementary density functional theory (DFT) calculations and experimental results (Figure 1c–f).^26,41−43^ On the other hand, the surface of the unpretreated lithium metal electrodes mainly consists of carbon and oxygen with pronounced C–H and C=O resonances. Furthermore, small amounts of silicon could be identified on the prepared pLi electrodes, probably originating from the silicon-coated Mylar foil used during the pressing of lithium (Figure S1).

**Figure 2:**
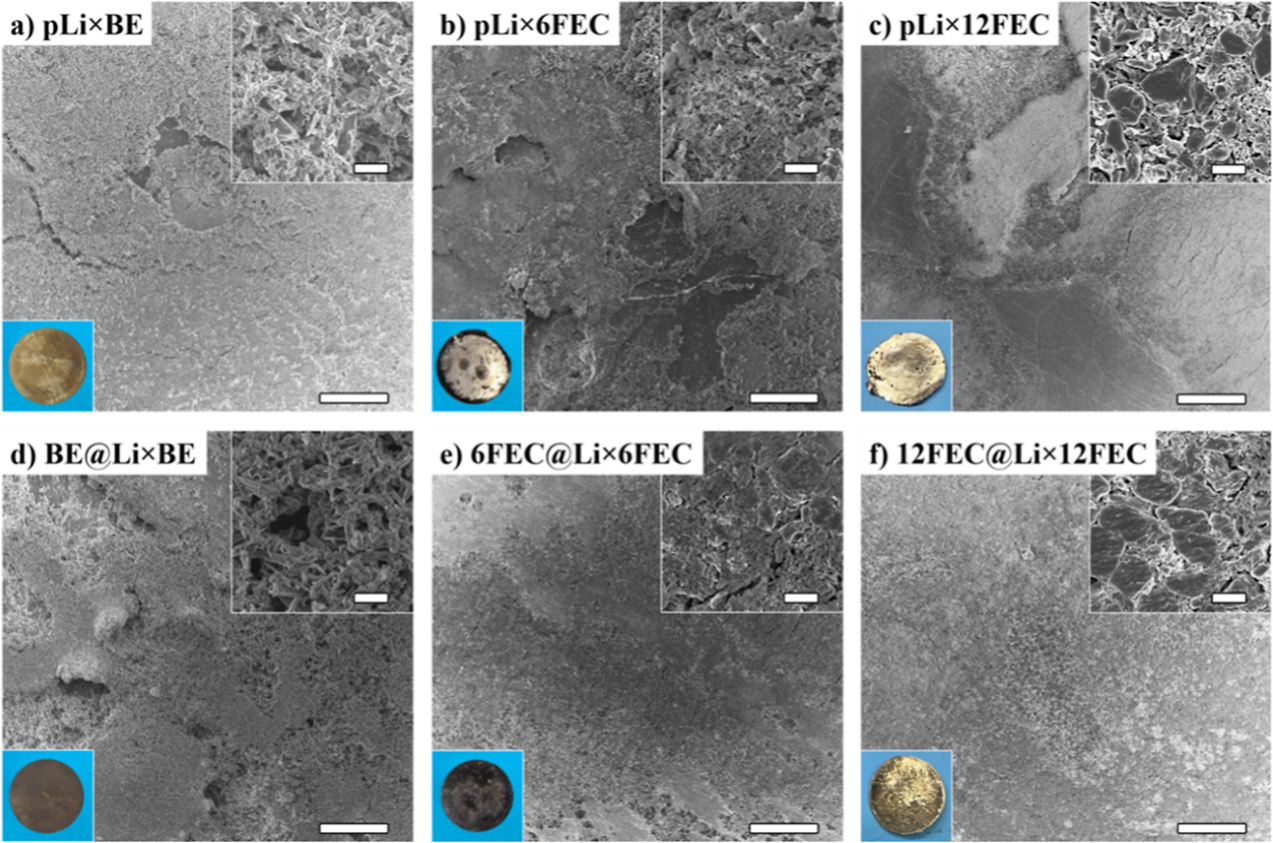
*Post mortem* SEM and optical images of the respective electrodes after 50 charge/discharge cycles at 0.5 mA cm^–2^ and a cycle duration of 2 h: (a) pLi × BE, (b) pLi × 6FEC, (c) pLi × 12FEC, (d) BE@Li × BE, (e) 6FEC@Li × 6FEC, and (f) 12FEC@Li × 12FEC. SEM scale sizes: 100 μm (big), 5 μm (small).

The surface analysis via SEM after 50 stripping/plating cycles revealed that with increasing FEC concentrations in the electrolyte, a dense surface morphology of the deposited lithium can be achieved on the pristine lithium electrodes (pLi) with pLi × 12FEC having a mosaic-like surface structure in contrast to the more mossy-like lithium deposits observed with pLi × BE (Figure 2). The generation of this dense lithium deposition morphology can be attributed to two properties induced by FEC: first, the decomposition of FEC results in the formation of LiF, which acts as a vital SEI component to protect the lithium anode from further parasitic side reactions and enables the uniform Li deposits.^27^ In addition, a four times faster SEI formation with a twice as fast Li^+^ ion exchange is achieved when FEC is present in an organic carbonate-based electrolyte, reducing stress-build up in the SEI during galvanostatic cycling.^32^

However, considerable parts of the Li metal surface seem to remain in a pristine state when cycled with FEC, as the main stripping/plating takes place on the edges of the electrode to avoid the stack pressure of the cell (Figure 2b,c). In the presence of a pSEI, a homogeneous Li stripping/plating on the whole lithium metal surface is achieved, and a dense mosaic-like surface morphology becomes observable with all of the FEC-containing electrolytes (Figure 2e,f).

Galvanostatic cycling of the cells containing the pretreated electrodes with the FEC-free BE electrolyte revealed a partial detachment of the pSEI taking place, with highly porous Li deposits emerging regardless of the presence and concentration of FEC during pretreatment (Figure S3). This indicates that unreacted FEC must be present in the electrolyte during cycling to achieve the mentioned dense mosaic-like lithium deposition structure.

**Figure 3:**
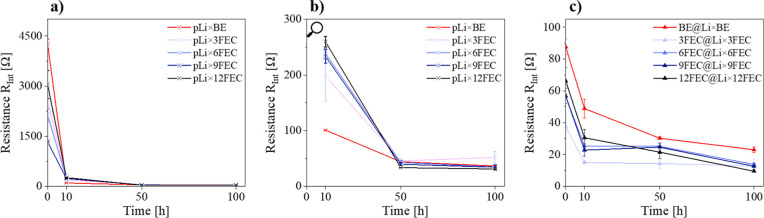
Interphasial resistances *R*_Int_ with (a) pLi electrodes before (0 h) and (b) pLi electrodes after 5, 25, and 50 stripping/plating cycles (0.5 mA cm^–2^, 1 h charge/discharge) and (c) pretreated electrodes. The interphasial resistances were obtained from their respective Nyquist plots fitted with the described equivalent circuit and averaged over at least three cells.

The positive impact of FEC in the resulting electrolyte formulation and the replacement of the NPL with a pSEI were further validated by EIS measurements (Figure 3). High initial resistances up to 4255 Ω were obtained with the pLi electrodes (Figure 3a), which can be mainly attributed to the high impact of the charge-transfer resistance (Figure S4), caused by the presence of the NPL.

Galvanostatic cycling of these cells led to a considerable decrease of the interphasial resistance after 10 h, most drastic on the pLi × BE probably caused by the expanded stripping/plating on the whole electrode and the resulting increased surface area observed in the SEM images of pLi × BE (Figure 3b). After an additional 90 h of cycling, *R*_Int_ further decreases and converges to ∼40 Ω, with pLi × 12FEC and pLi × 9FEC displaying the lowest resistance values. This explains why the Li stripping/plating only occurs on small areas of the lithium electrode cycled with FEC because as soon as parts of the very resistive NPL are replaced by a highly ion-conductive FEC-induced SEI, lithium release and deposition on these areas are strongly favored.

If the pretreated electrodes are used, the high initial resistance can be avoided and a resistance decrease of ∼95% is achieved, mainly caused by the absence of any significant charge-transfer contribution observed (Figure S5). The presence of FEC during electrode pretreatment leads to a further decrease in the interphasial resistance at first with no correlation of the FEC concentration (Figure S6). This positive effect of the FEC-derived pSEI on the interphase resistance diminishes after 50 h of cycling with BE, as no distinct difference in *R*_Int_ between BE@Li × BE and 3/6/9/12FEC@Li × BE is visible. By adding FEC to the electrolyte, even lower *R*_Int_ down to 10 Ω can be achieved for 12FEC@Li × 12FEC (Figure 3c), further underlining the importance of FEC in the electrolyte during galvanostatic cycling.

Notably, the influence of the NPL on the interphasial resistance is still present even after 100 h of cycling, as the electrodes with an NPL still have roughly three times the interphasial resistance than their counterparts with a pSEI at that point in time.

#### 3.1.1 Electrochemical Performance Evaluation

**Figure 4:**
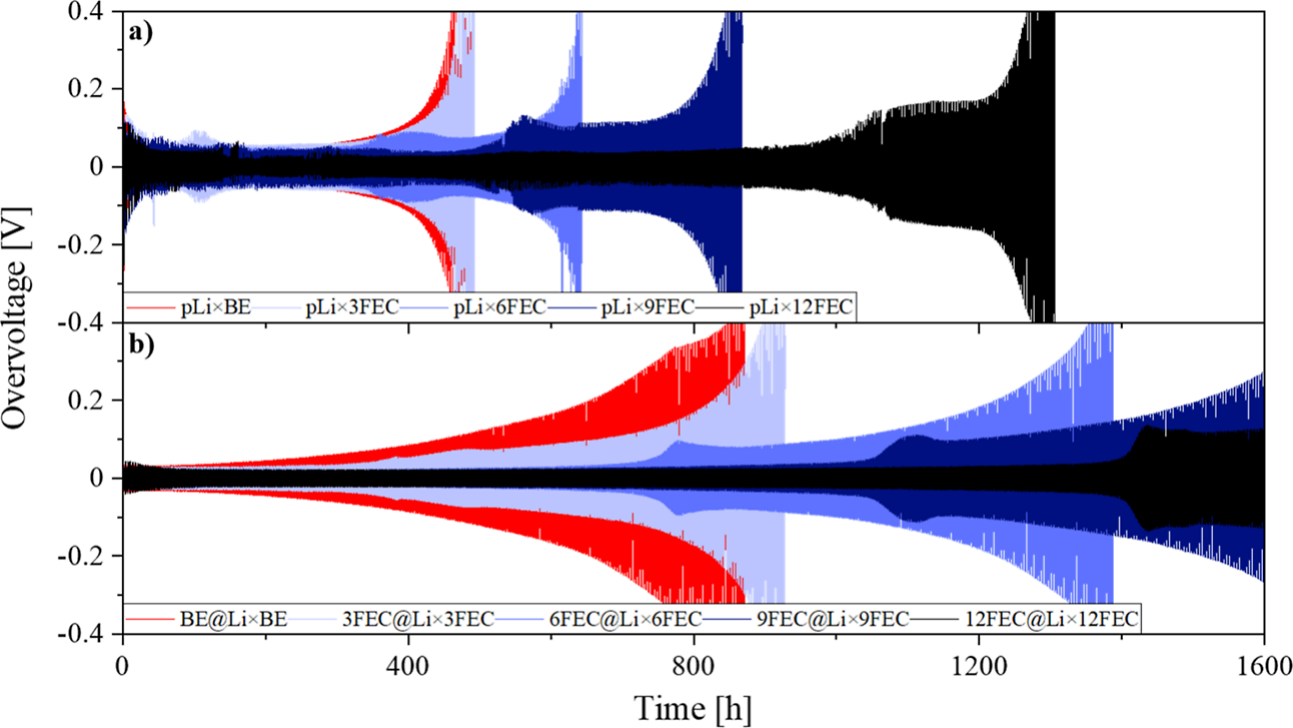
Overvoltage profiles in symmetric Li||Li coin cells (0.5 mA cm^–2^, 1 h charge and discharge) with (a) the unpretreated electrodes and (b) the pretreated Li metal electrodes with the respective electrolyte formulation.

To investigate the effect of FEC concentration and the introduction of an FEC-derived pSEI on long-term performance, symmetric Li||Li was galvanostatically cycled and analyzed (Figure 4). The cells cycled with pLi electrodes generated a high initial overvoltage of roughly 0.3 V, which aligns with the observations from the EIS measurements that revealed a very resistive nature of the NPL covering the pristine lithium (Figure 4a). The continuous rupture of the NPL and the replacement with a less resistive in situ formed SEI leads to a decline of the initial overvoltage down to 0.05 V for the cell setups pLi × BE (red), pLi × 3FEC (light blue), pLi × 6FEC (blue), and pLi × 9FEC (dark blue) in the first 50 h during cycling. With the FEC-rich 12FEC electrolyte (black), an overvoltage of only 0.02 V can be achieved, however, which is accompanied by a more inhomogeneous overvoltage evolution during galvanostatic cycling. In accordance with previous research, the presence of FEC prolongs the lifetime of the resulting cell as the smooth and thin FEC-induced SEI decelerates the continuous electrolyte consumption, which is eventually the cause of the rising overvoltages and the death of the cell.^26,27^ Due to the constant reformation of the SEI accompanied by continuous consumption of FEC, higher concentrations of the film-forming additive/cosolvent naturally prolong the lifetime of the corresponding cell. The presence of 3FEC only delays the predetermined cell failure at 0.4 V overvoltage by 10 h compared to BE, whereas the electrolytes 6FEC, 9FEC, and 12FEC can increase the cycle life by 170, 380, and 805 h, respectively.

With the introduction of a pSEI, a further enhancement of the cell lifetime by 85% for BE and ∼100% for 3FEC and 6FEC can be achieved (Figure 4b). For 9FEC and 12 FEC, a similar trend with respect to the cycle life can be expected; however, the respective cells do not exceed the 0.4 V overvoltage threshold within the observation period of 1600 h of cycling. The cracking and replacement of the NPL observed in the *post mortem* SEM and EIS analysis are responsible for a considerable amount of the electrolyte consumption and by omitting this *in situ* formation step with the pSEI, the electrolyte-derived cell death can be delayed. The absence of the resistive NPL also prevents the high initial overvoltages observed for the pLi cells and, thus, homogeneous overvoltage profiles with plateaus at as low as 0.02 V for 6FEC, 9FEC, and 12FEC can be achieved right from the beginning. As observed during the EIS measurements, the influence of the NPL is not only limited to the first cycles but has a lasting negative impact on the interphasial properties of Li discernible on the high overvoltages during galvanostatic cycling. Employing the different FEC-induced pSEIs with the BE electrolyte, no difference in the overvoltage profiles or the cell lifetime compared to BE@Li × BE can be observed further emphasizing the importance of FEC in the electrolyte during cycling (Figure S7). Only with 12FEC@Li, a prolonged cycle life can be achieved, which is, however, accompanied by an inhomogeneous overvoltage behavior.

**Figure 5:**
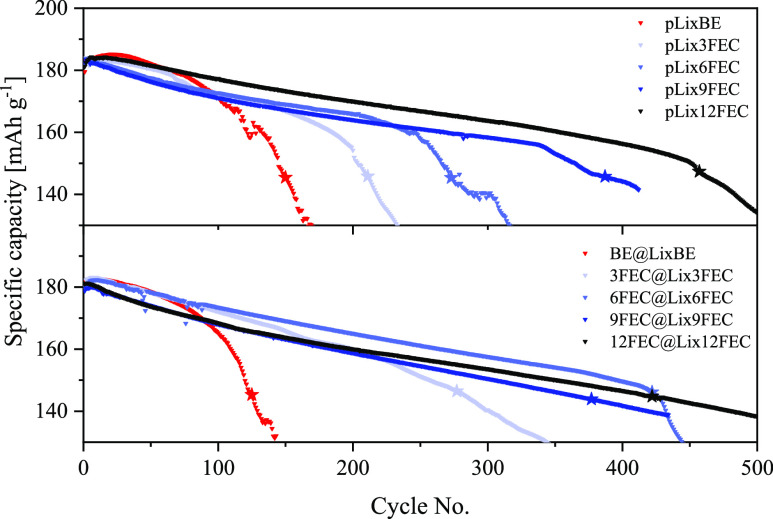
Specific capacity profiles of the NMC811||Li cells at a constant current density of 0.5 mA cm^–2^ (∼0.5C) between 3.0 and 4.2 V with (a) the pLi electrodes and (b) the pretreated electrodes with the respective electrolyte formulation used for the pretreatment.

The positive impact of FEC in the electrolyte further translates to the NMC811||Li full-cell setup. The FEC-free cell setup pLi × BE experiences the fastest capacity decline and reaches the 80% State-of-Health (SOH) threshold, which is marked with a star, after 150 cycles (Figure 5a, red). With the addition of FEC, a less drastic linear decline can be achieved, followed by a capacity drop that is delayed to higher cycle numbers with higher concentrations of FEC. This behavior was also found in Si-based cells as NMR spectroscopy revealed a correlation between the total consumption of FEC and the onset of the capacity drop.^26^ As a consequence, 12FEC (black) can achieve the most extended cell lifetime with 458 cycles, followed by 9FEC (dark blue), 6FEC (blue), and 3FEC (light blue) with 388, 272, and 211 cycles, respectively. Nonetheless, in the first 61 cycles, the FEC-free setup actually has a higher specific discharge capacity as the specific capacity of the cell slightly increases until the 21st cycle before a rapid capacity decline begins. This anomaly is assumed to be caused by an initial, thinner but less effective SEI, formed in the presence of the BE electrolyte, which causes less polarization in the beginning and thus leads to higher capacities during the first cycles compared to the FEC-containing cell setups.^24^ Based on the EIS data, which showed a less resistive interphasial layer with BE after the first cycles compared to the FEC-containing cell setups, a similar assumption can be made for the NMC811||Li cells.

Coupling the pretreated electrodes with the NMC811 electrode, a further delay of the capacity drop with the FEC-containing electrolytes can be achieved, probably due to the decelerated FEC consumption observable in the corresponding symmetric Li||Li cells (Figure 5b). This prolongs cycle life by 67 and 152 cycles for the 3FEC- and 6FEC-based cells, respectively. Even though a capacity drop was avoided for cells containing 9FEC@Li × 9FEC and 12FEC@Li × 12FEC, a prolonged cell lifetime could not be achieved due to a higher capacity decline in the first cycles compared to their unpretreated counterparts. A similar phenomenon is observed for BE@Li × BE, which does not experience the rising capacity retention in the first cycles as pLi × BE and thus reaches its predetermined death 26 cycles earlier, probably since a thicker, more effective SEI is already present during the first cycles. Similar to the symmetric cells using the FEC-pretreated electrodes with BE, no beneficial effects are observable, as the cell deaths occur at similar cycle numbers (Figure S8). Only 12FEC@Li × BE shows a slight increase in the cycle life, which might be due to FEC residues in or on the pSEI which is underlined by the FEC-specific capacity drop.

## 4 Conclusions

In this work, an in-house-designed and developed lithium pretreatment method has been utilized to systematically evaluate the effect of varying FEC concentrations on the interphase properties and its impact on the galvanostatic cycling performance of lithium–metal batteries. The presence of FEC as a functional additive/co-solvent in the organic carbonate-based electrolyte leads to a dense mosaic-like lithium deposition morphology with a reduced polarization of down to 20 mV. In addition, the cycle life of symmetric Li||Li and NMC811||Li cells could also be successfully prolonged with increasing concentrations of FEC in the considered electrolyte formulation. If the FEC is solely present in the formation of the pSEI, the observed benefits of the additive with regard to the interphase properties and the cycling performance could not be achieved even though the surface analysis by XPS revealed a LiF-rich decomposition layer on the lithium electrode. By combining the FEC-containing electrolyte with the FEC-derived pSEI, the positive effects of the FEC could be even more enhanced in comparison to those of the cells with the pristine lithium electrodes.

This systematic study has revealed the importance of FEC in the electrolyte during galvanostatic cycling to achieve the benefits associated with the enhancement of the LMB performance. The mere existence of a LiF-rich FEC-derived SEI is not advantageous if no FEC is present to maintain the interphase during galvanostatic cycling.

## Supporting Information Available

Additional experimental data, including SEM images and electrochemical analysis of the pretreated electrodes cycled with the additive-free electrolyte BE (PDF)
