# Supplementary material for: Tailoring the Preformed Solid Electrolyte Interphase in Lithium Metal Batteries: Impact of Fluoroethylene Carbonate
Source: ACS Appl Mater Interfaces. 2023 Nov 8;15(46):53526–32. doi: 10.1021/acsami.3c12797 (PMC10685346; doi:10.1021/acsami.3c12797)
Supplement: Supplementary file 1 — am3c12797_si_001.pdf [file am3c12797_si_001.pdf]

Supporting Information

"Tailoring the Preformed Solid Electrolyte Interphase in Lithium-Metal Batteries: Impact of Fluoroethylene Carbonate"

Dominik Weintz[a], *Sebastian P. Kühn*[a], *Martin Winter*[a,b], *Isidora Cekic-Laskovic*[*,a]

[a] Forschungszentrum Jülich GmbH, Helmholtz-Institute Münster (IEK-12), Corrensstrasse 48, 48149 Münster, Germany

[b] University of Muenster, MEET Battery Research Center, Corrensstraße 46, 48149 Muenster, Germany

Corresponding Author

*E-Mail i.cekic-laskovic@fz-juelich.de

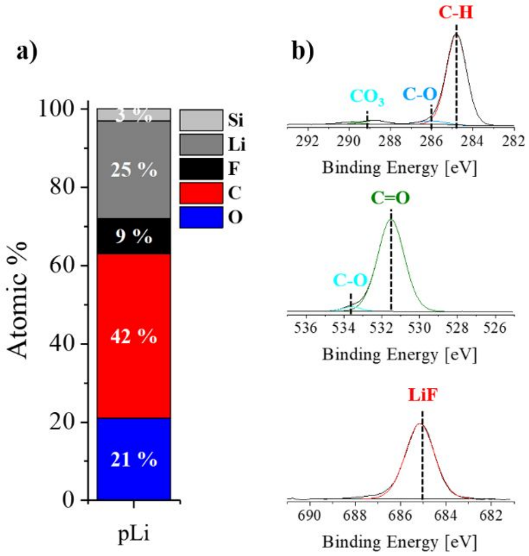

Figure S1. XPS analysis of the pristine lithium electrode. a) Atomic distribution of O, C, F, Si and Li; b) Chemical resonance with the focus on F 1s, O 1s and C 1s.

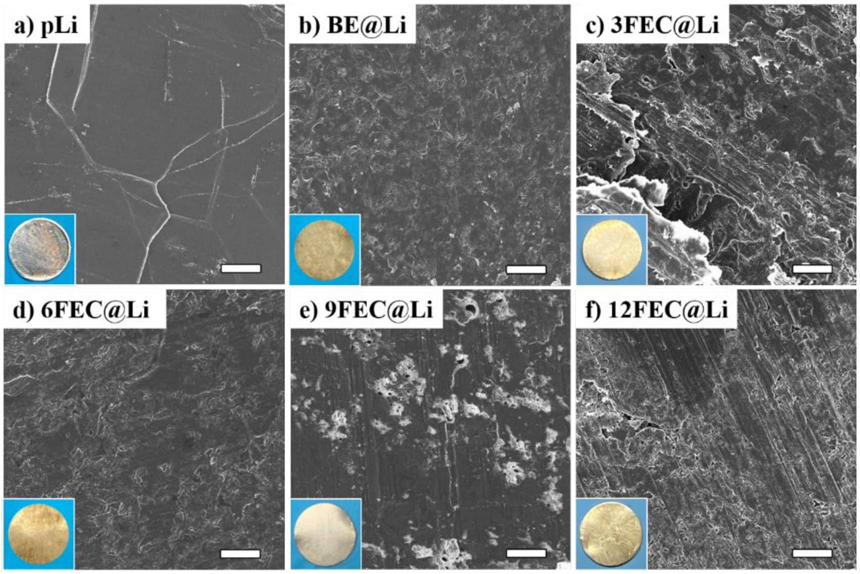

Figure S2. Optical and SEM images of the surface of pristine lithium pLi (a), as well as of the pSEI on BE@Li (b), 3FEC@Li (c), 6FEC@Li (d), 9FEC@Li (e) and 12FEC@Li (f).

SEM scale size: 10 μm

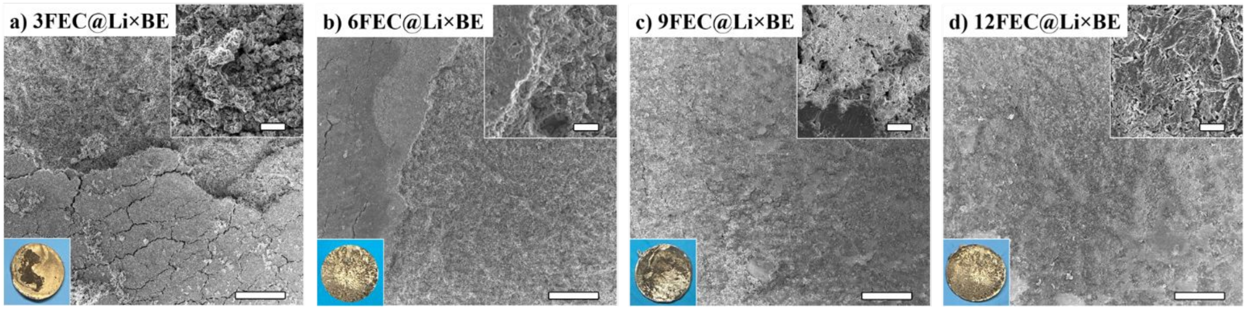

Figure S3. *Post mortem* SEM images and optical pictures of the respective Li metal electrodes after 50 cycles at 0.5 mA cm^{-2} with a cycle duration of 2 h: a) 3FEC@Li×BE, b) 6FEC@Li×BE, c) 9FEC@Li×BE and d) 12FEC@Li×BE.

SEM scale sizes: 100 µm (big), 5 µm (small)

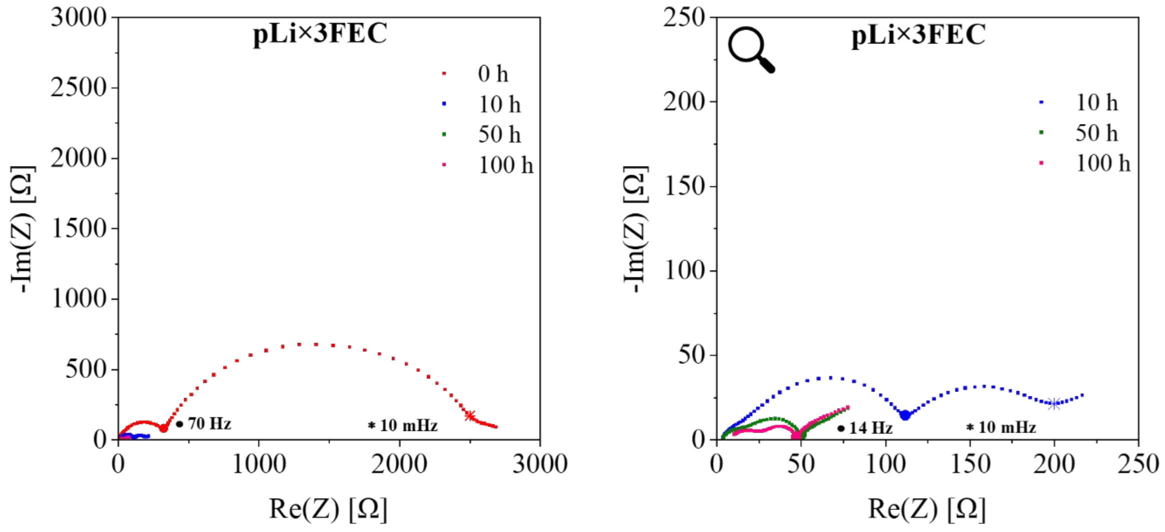

Figure S4. Nyquist plot of the cells containing pLi×3FEC before cycling (0 h) as well as after 10 h, 50 h and 100 h of galvanostatic cycling (0.5 mA cm^{-2}, 2 h cycle duration).

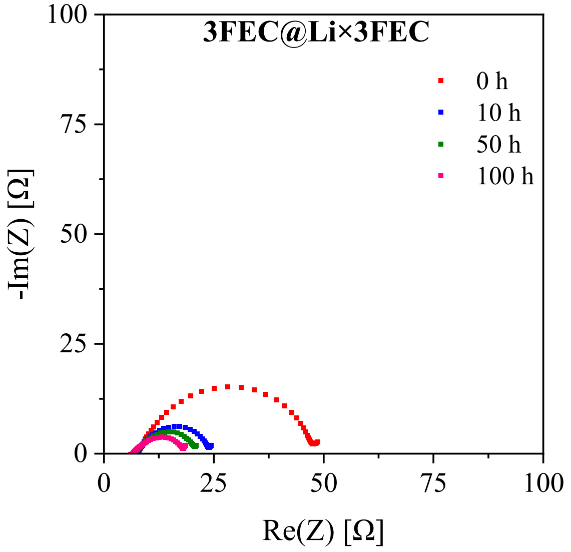

Figure S5. Nyquist plot of the cells containing 3FEC@Li×3FEC before cycling (0 h) as well as after 10 h, 50 h and 100 h of galvanostatic cycling (0.5 mA cm^{-2}, 2 h cycle duration).

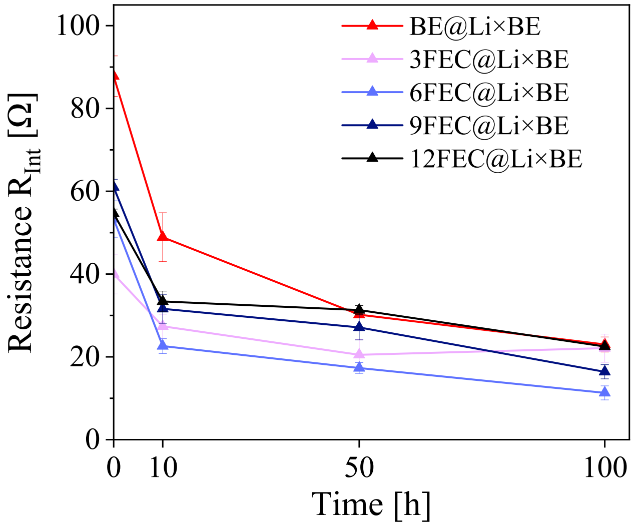

Figure S6. Interphasial resistance R_{Int} profiles of the cells containing the pretreated Li metal electrodes and BE as an electrolyte before (0 h) and after several stripping/plating cycles (0.5 mA

cm^{-2}, 2 h cycle duration). The interphasial resistances were obtained from their respective Nyquist plots fitted with the described equivalent circuit and averaged over at least three cells.

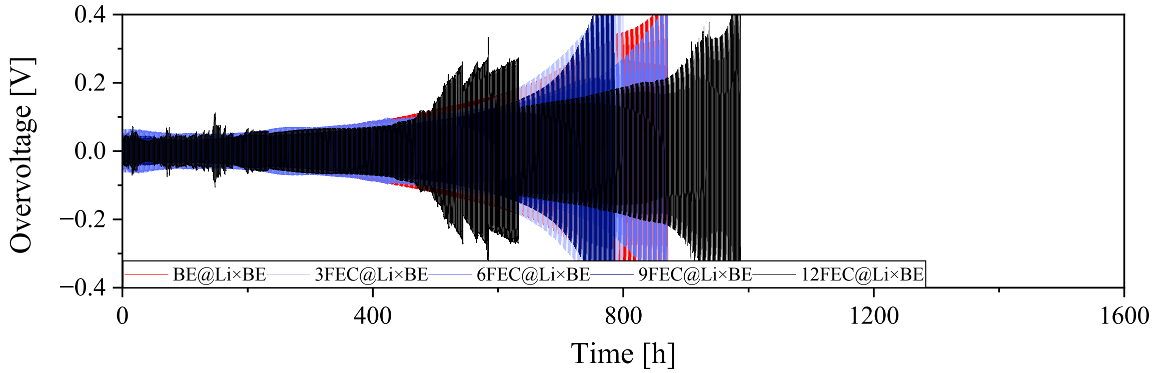

Figure S7. Overvoltage profiles in symmetric Li||Li coin cells (0.5 mA cm^{-2}, 1 h charge and discharge) with the pretreated Li metal electrodes and the electrolyte BE.

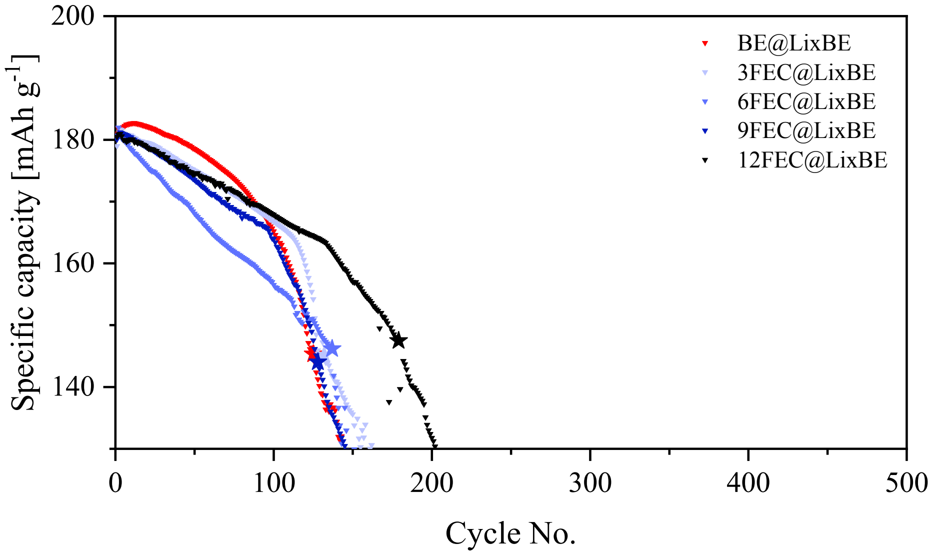

Figure S8. Specific capacity profiles of the NMC811||Li cells at a constant current density of 0.5 mA cm^{-2} (~0.5 C) between 3.0 V and 4.2 V with pretreated Li metal electrodes and BE electrolyte.
